# Blood-based liquid biopsies for prostate cancer: clinical opportunities and challenges

**DOI:** 10.1038/s41416-022-01881-9

**Published:** 2022-06-17

**Authors:** Blanca Trujillo, Anjui Wu, Daniel Wetterskog, Gerhardt Attard

**Affiliations:** University College of London. UCL Cancer Institute, Paul O’Gorman Building, 72 Huntley st, London, WC1E 6BT UK

**Keywords:** Prostate cancer, Scientific community

## Abstract

Liquid biopsy has been established as a powerful, minimally invasive, tool to detect clinically actionable aberrations across numerous cancer types in real-time. With the development of new therapeutic agents in prostate cancer (PC) including DNA repair targeted therapies, this is especially attractive. However, there is unclarity on how best to screen for PC, improve risk stratification and ultimately how to treat advanced disease. Therefore, there is an urgent need to develop better biomarkers to help guide oncologists’ decisions in these settings. Circulating tumour cells (CTCs), exosomes and cell-free DNA/RNA (cfDNA/cfRNA) analysis, including epigenetic features such as methylation, have all shown potential in prognostication, treatment response assessment and detection of emerging mechanisms of resistance. However, there are still challenges to overcome prior to implementing liquid biopsies in routine clinical practice such as preanalytical considerations including blood collection and storage, the cost of CTC isolation and enrichment, low-circulating tumour content as a limitation for genomic analysis and how to better interpret the sequencing data generated. In this review, we describe an overview of the up-to-date clinical opportunities in the management of PC through blood-based liquid biopsies and the next steps for its implementation in personalised treatment guidance.

## Introduction

In the UK, prostate cancer is the most common cancer in men with approximately 48,500 cases diagnosed every year. With 11,855 prostate cancer-related deaths annually [1], it is a major healthcare and economic burden. Prostate cancer is a heterogeneous disease that is characterised by high variability in clinical outcomes. There is therefore an urgent clinical need to use new tools to enable better screening programmes, improve risk stratification at clinical decision points and select the most effective treatment that maximise cure and extend life expectancy, whilst minimally altering patients’ quality of life.

Molecular profiling of solid cancers has been used across multiple cancer types to identify poorer prognosis cancers and guide treatment selection. Most studies have utilised analysis of DNA and/or RNA and/or protein on a biopsy from the primary tumour or less commonly, a metastatic lesion [2]. There are some major limitations with this approach. First, there are practical and clinical challenges to obtaining tissue from poorly accessible metastases and primary samples are archived formaldehyde-treated. The latter is especially true for prostate cancer as up to 90% of patients have metastases to the bone [3]. Secondly, intra-patient and tumour heterogeneity may result in a missed or incorrectly classified cancer, due to spatial or temporal differences. Third, repeated tumour biopsies to monitor tumour evolution and response dynamics is not feasible.

**Fig. 1:**
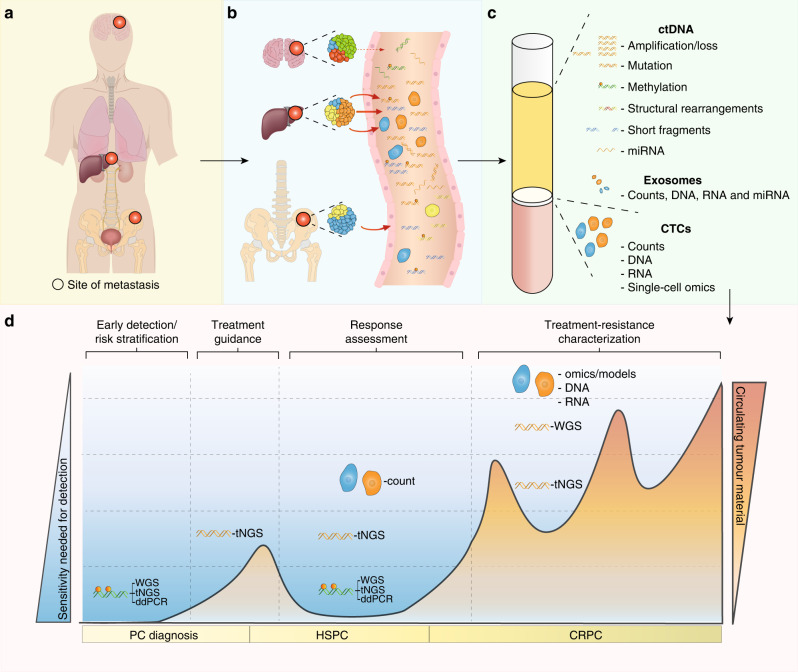
Liquid biopsy in prostate cancer: tumour features, clinical utility and challenges. **a** Example of actively proliferating prostate cancer metastatic sites. **b** Potential differential tumour material release with the highest contribution from the liver metastasis followed by the bone and restricted release from the brain due to the blood/brain barrier. **c** Circulating tumour material features that can be isolated and analysed from blood. **d** Potential clinical utility of liquid biopsy analysis in prostate cancer management from diagnosis to death and listing the most suitable methods for analysis in each set to overcome the respective sensitivity requirements. ctDNA circulating tumour DNA, miRNA microRNA, CTCs circulating tumour cells, tNGS targeted next-generation sequencing, WGS whole-genome sequencing, ddPCR droplet digital PCR, PC prostate cancer, HSPC hormone-sensitive prostate cancer, CRPC castration-resistant prostate cancer.

Due to these limitations, there has been an interest in blood-based biomarkers, commonly referred to as liquid biopsies, in which we focus on this review, as an alternative or companion to solid tumour biopsies and imaging studies to better characterise tumour molecular drivers and response to treatment. Nucleic acids such as DNA or RNA as well as proteins, cells and vesicles circulate freely in human blood and can be isolated using various molecular techniques (Fig. 1). Circulating cell-free DNA (ccfDNA), probably now the most studied circulating molecule, was first clinically implemented in prenatal diagnostics to detect congenital disorders [4]. In blood, ccfDNA is naturally fragmented to a size of 142–170 base pairs and where the fragment sizes relate to the length of DNA wrapped around nucleosomes and the subsequent cleavage of unprotected DNA between nucleosomes by nucleases [5]. Tumour-derived ccfDNA is also known as circulating tumour DNA (ctDNA). The ctDNA fraction of the total ccfDNA depends on the disease setting and tumour spread and can range from 1% or below at diagnosis and/or in patients with localised disease, and up to 90% in patients with high-volume progressing metastases in castration-resistant prostate cancer [6–8]. Intact tumour cells, named circulating tumour cells (CTC) when in blood, can also be isolated and are shed by the tumour into the bloodstream. Compared to white blood cells, CTCs are present in very low abundance (approximately fewer than 10 cells/mL of blood in metastatic patients) [9]. This makes them challenging to detect and isolate [9].

Liquid biopsies also have inherent limitations. CTC-based mRNA and protein analyses are underdeveloped and usually unimodal (one or fewer markers studied) and limited to the characterisation of very small numbers of CTCs. Thus, CTC derived tumour genome representation can be far less than that usually derived from tumour biopsies [10]. ctDNA analysis is performed in a background of a potentially large amount of DNA derived from normal cells and on a mixture of material from multiple tumour clones that could be differentially represented in circulation due to variable rates of release of material into circulation. This might not necessarily be representative of their aggressiveness, but rather by the metastatic site (for example, liver versus brain metastases) or by histology as seen for lung squamous cell carcinomas which associate with higher ctDNA levels than lung adenocarcinomas [11] (Fig. 1).

Other body fluid components, such as urine, have also been subjected to DNA extraction and NGS analyses with promising studies reported in the field [12–14].

## Preanalytical considerations in liquid biopsies

To maximise the reliability of molecular information obtained from a liquid biopsy, there is a need to develop a standardised workflow for the preanalytical steps. Different approaches for collecting, processing and storing blood can lead to an up to 50% variability in the amount of ccfDNA extracted [15]. Each of these steps could affect the analytical outcome of certain assays resulting in different study results [16, 17].

For cfDNA analysis, one of the key preanalytical goals is to avoid genomic DNA release from lysing leucocytes: this DNA would dilute the already potentially small fraction of tumour-derived DNA and can lead to false-negative results [18]. In order to prevent this, blood collected in EDTA-K3 tubes, arguably the gold standard, must be processed within 2 h after venepuncture [5]. To allow greater flexibility for clinical implementation, blood collection tubes (BCT) containing fixative agents have been developed. These aim to prevent cell membrane lysis and can be processed 7 days or longer after blood draw. These tubes are now widely used, although some contain formaldehyde-like chemicals which cause cross-linking and chemical modification, potentially interfering with DNA amplification and downstream assessments such as methylation ratios [6].

Pre-analytic processing of samples after blood draw should be carefully optimised, for example extending the time of proteinase K incubation of plasma during DNA extraction can minimise cross-linking-related artefacts due to the presence of fixatives in BCTs [2].

Another important preanalytical goal is to achieve the highest extraction efficiency for fragments of ccfDNA below 200 bp as this range contains most ctDNA fragments. Different studies have tested several extraction methods showing differences in the percentage of short ccfDNA isolated depending on the kit used [19].

For CTCs analysis, different preanalytical steps should be considered. Firstly, for blood collection, preservatives are needed to avoid cell lysis although results can be contradictory depending on the BCT used [20]. New approaches that aim for cell viability are able to maintain viable cells after 6 days of storage [21]. Secondly, nucleic acid isolation and amplification from CTCs remains a challenge with different methods prone to errors to identify genetic variations [22, 23].

Extracellular vesicle (EV) collection represents a particular challenge as vesicle release from blood cells (especially platelets) after venepuncture needs to be minimised to avoid reduction in purity. However, to date, no specific EV preservative is commercially available [24, 25]. Widely used isolation methods like ultracentrifugation do not distinguish between different subsets of EVs (exosomes, microvesicles) [26] and others like immunoaffinity methods (Miltenyi^®^), which target different proteins (CD81, CD63), are capable to separate EVs but are expensive [27]. Currently, no consensus has been agreed on which method results in a higher-quality yield [28].

## Plasma-derived nuclei acid features and their utility in prostate cancer management

### Genome

ctDNA analysis of metastatic prostate cancer patients is able to identify prostate cancer genomic features. When the circulating tumour fraction is sufficiently high there is a high concordance with tissue findings for truncal alterations present in concurrently collected biopsies of metastases [29, 30]. However, low-abundance alterations private to sub-clones of cells can be missed, especially at lower tumour fractions [11].

Techniques such as digital droplet PCR (ddPCR) and similar targeted approaches have been widely used and can capture a handful of mutant alleles at very high sensitivity (0.001%) [31]. This can allow cost-effective testing of pre-defined mutations of relevance in specific clinical scenarios.

However, it is the development of next-generation sequencing (NGS) what has enabled molecular characterisation of cancer at a reasonable cost. These include the possibility of whole-genome sequencing (WGS), whole-exome sequencing (WES) or targeted sequencing. Whole-genome libraries can be enriched to analyse specific DNA targets mainly by two approaches: amplicon-based or hybridisation-based method. Amplicon-based uses pre-designed primers to amplify regions of interest by PCR and covers generally a limited panel of mutations whereas the hybridisation capture method allows for larger size panels and uses biotinylated probes (oligonucleotides complementary to regions of interest) that bind to PCR-amplified DNA and are isolated by streptavidin-coupled magnetic beads. These targeted approaches have been reported to detect prostate cancer mutations in blood and are used in clinically validated tests such as the UW-Oncoplex (UW-OncoPlex^TM^) and the Foundation Liquid CDx (FoundationOne^®^) test [7, 8, 32, 33]. The main limitation in using a targeted panel is the risk of missing relevant driver alterations. This can be addressed with tumour informed design of patient-specific panels, but this is not currently feasible [11]. The advantage of targeted sequencing is the opportunity to achieve high coverage across regions of interest and needs to be balanced against a broader approach that achieves lower coverage.

The overall sensitivity for detection of an alteration is dependent on the tumour content (TC) of ccfDNA. This could be generated either as a measure of the most abundant alteration detected or using a bespoke orthogonal assessment. The most effective methods for measuring TC include commonly occurring clonal events. In situations such as metastatic breast cancer where a discrete number of hot-spot mutations are commonly clonal, restricted assays have been used [11, 34]. Patient-specific probe sets that track clonal mutations detected in multi-region sequencing of patient tumours have been performed in non-small cell lung cancer [11]. Prostate cancer is mainly characterised by recurrent copy number changes rather than recurrent mutations in comparison to other cancer types [35, 36]. Approaches that leverage pan-genome or recurrent copy number alterations could therefore be best suited to derive a measure of whether ctDNA is present and potentially at what abundance [7, 8, 37, 38]. Most of these approaches reported to date have a lower limit of sensitivity of 5–10%, which detects tumour in the majority of patients with progressing metastatic disease but may not be sufficiently sensitive in patients with low-volume metastases. In prostate cancer, detection of ctDNA is prognostic and a change in ctDNA with treatment associate with the differential outcome with treatment [37, 39].

Once prostate cancer relapses after castration, the most common genomic events that emerge involve the androgen receptor (*AR*) [7, 8, 40]. In particular, *AR* is typically amplified or mutated in this setting following the selective pressures from androgen deprivation therapy (ADT) [7, 8, 41]. Two *AR* point mutations emerge after treatment with abiraterone (combined with prednisone) and appear to be the most commonly detected in metastatic castration-resistant prostate cancer (mCRPC), *AR* L702H (*AR c.2105* *T* > *A*) occurs primarily in patients previously treated with glucocorticoids and *AR* T878A (*AR c.2632* *A* > *G*) is associated with progesterone-mediated activation. Detection of *AR* gene alterations in circulation associate with worse outcome in patients treated with androgen receptor signalling inhibitors (ARSI) such as abiraterone or enzalutamide [42]. A number of other aberrations are prognostic in multivariate analyses when detected prior to treatment, including *TP53, RB1* and *PTEN* [37, 43]. In non-randomised cohorts, it is challenging to separate the effect of the alteration on worse outcome independently from the increased chance of detecting the alteration with higher tumour fraction. Further work is required to determine the clinical relevance of detection of these alterations prior to a treatment. On treatment, patients who show a loss of detection or drop in the abundance of ctDNA after 4–8 weeks treatment have similar outcomes to patients with no detection prior to treatment; in contrast persistent or appearance of ctDNA on treatment is associated with the worst outcomes [37].

Following the recent approval of PARP inhibitors for mCRPC, ctDNA assessment has been clinically approved for detection of alterations in DNA damage response genes (DDR). DDR genes are aberrant in up to 20% of mCRPC, most commonly *BRCA2* (13%) [44]. Gene loss or loss-of-function mutations in *BRCA2* associate with more aggressive disease and if detected in tumour or plasma, can be used to select patients for PARP inhibition [43]. Although PARP inhibitor sensitivity is dependent on the functional loss of both alleles, evidence of a disruptive alteration in one allele in circulation is often accepted as sufficient evidence to offer a patient PARPi treatment [45]. In addition, 2–3% of prostate cancer harbour a deficient mismatch repair gene (dMMR) or microsatellite instability (MSI) with reports of durable responses to immune checkpoint inhibitors [46]. Finally, an especially aggressive subset of mCRPC (currently estimated at 15–20% of advanced prostate cancers) develop an AR-independent phenotype that can associate with neuroendocrine carcinoma histologic features on tissue biopsy. This phenotype also appears to be characterised by a higher prevalence of loss of tumour suppressor genes such as *TP53* and *RB1* [47]. These alterations all represent potential future biomarkers for treatment selection.

In conclusion, whilst ctDNA genomic analysis is now implemented in the clinical management pathway and will be increasingly used by physicians, technical improvement in current assays and combining with other data will improve the granularity of the read-out and the patient populations liquid biopsies can be applied to.

### Methylome

Epigenetic changes cover functionally relevant changes to the genome that do not involve alterations in the DNA sequence. They are mainly involved in gene expression modification. The most well-studied epigenetic feature of cfDNA is 5-methylcytosine (5mC) at cytosine–guanine (CpG) sites that tend to cluster in CpG islands in the promoter region of genes and are widely known to be involved in gene silencing [48, 49]. DNA methylation patterns are tissue-specific and cfDNA methylation landscape contains a mixture of methylation signal from various tissue types. Traditionally most used approach is to employ differential methylation analysis (DMR or DMP) with selected FDR cut-off [50–52]. Several studies have leveraged this for cell fraction deconvolution based on either supervised or semi-supervised mathematical approach and reference tissue database [51, 53–55]. Selection of a valid ctDNA methylation marker must ensure high clonality, low inter-individual variability and tumour and tissue specificity. Genome-wide methylation changes occur over the course of prostate cancer initiation, progression and metastases. These recurrent and abundant events, occurring across large genomic regions, could be exploited to address some of the limitations of genomic testing for prostate cancer, such as low tumour content [56–60]. Large-scale studies have established the paradigm that ctDNA methylation profiling has superior cancer detection sensitivity and tissue-of-origin specificity than genomic approaches such as whole-genome or targeted sequencing [61].

There are a number of recurrent DNA methylation alterations, such as *GSTP1, APC, RASSF1A*, with potential transcriptomic or physiologic impacts on prostate cancer [62]. Hypermethylated *GSTP1*, the most well-studied, aberrantly methylated gene in prostate cancer, is one of the earliest and most frequent events [63]. Given its frequent presence in prostate cancer tumorigenesis, several studies suggest *GSTP1* silencing to have a key role in tumour development [63–65]. Furthermore, *GSTP1* hypermethylation in tissue is prognostic in localised disease. In blood, several studies that used detection of *GSTP1* as a proxy for prostate tumour in blood have found this to be prognostic in relapsed patients [66, 67]. Analysis of multiple tissue-specific methylation markers using methylation array-based approach has shown ctDNA can be prognostic of treatment response in metastatic prostate cancer patients treated [54, 68, 69]. The limitation of methylation array is DNA input and restricts data quality in low-input ctDNA sample.

Whole-genome methylation assessment identifies 1000 s of regions that are differentially methylated in prostate cancer DNA compared to normal or leucocyte DNA [70]. This introduces an opportunity for including methylation-based information from a larger number of regions for more accurate assessment of ctDNA. Additionally, some studies have also extracted 5-hydroxymethylcytosine (5-hmc) information at regions that distinguishes cancer from non-cancer DNA and inform on underlying tumour biology [71].

Moreover, the aforementioned neuroendocrine phenotype is characterised by unique methylation patterns that can be detected in liquid biopsies analysing cfDNA and matching prostate tumour biopsies. This introduces the opportunity of early identification and avoids unnecessary biopsies in advanced prostate cancer patients [72].

### Fragmentome

Another potentially informative molecular feature of cfDNA is the DNA fragment length. The fragment length (a.k.a. fragmentomics) distribution of cfDNA, usually peaking at 167 bp, carries the information of nucleosome positioning which can be used to derive tissue-of-origin. As reported, cfDNA show different fragmentation patterns in tissue-specific open chromatin regions which relates to nucleosome positioning and reflects differences in sequencing coverage [73, 74].

In addition, variability of genome-wide plasma DNA coverage related to nucleosome occupancy can be used to infer gene expression patterns: cancer histological types could be differentiated based on their transcription factor accessibility patterns and nucleosome positioning which are both tissue-specific and different between healthy and cancer individuals. This approach could also identify re-programming of regulatory pathways in real-time, for example, the transition of AR-dependent prostate adenocarcinoma to an AR-independent “neuroendocrine” sub-type with distinct activated regulatory pathways [75].

ctDNA (derived from tumours) has been suggested to have shorter fragment length than associated non-tumour ccfDNA [76–78]. As a result, fragment size selection could allow enrichment of ctDNA, improving the detection of genomic alterations and tumour specific features at low tumour fractions [79].

## Transcriptome and protein in CTCs and exosomes

The study of RNA in liquid biopsies is hampered by the short half-life of messenger RNA in circulation. Therefore, efforts have focused on characterising RNA from isolated CTCs, extracellular vesicles and/or the study of short microRNAs (miRNA).

CTCs analysis is challenging due to the low ratio of cells compared to leucocytes (1 to several million) but adequate isolation and/or enrichment (often expensive) can allow for DNA, RNA and protein analysis plus single-cell assessment or in vitro culture studies [80, 81].

The number of CTCs detected in blood has been associated with treatment outcomes and overall survival in prostate cancer and other cancers [82]. CellSearch^®^ (currently distributed by Menarini Silicon Biosystems Inc.) was cleared by the FDA for prognostication of patients with advanced cancer using enumeration. It uses an immunomagnetic beads-based approach targeting EpCAM (epithelial cell adhesion molecule) present on the surface of most differentiated CTCs arising from adenocarcinomas. CTCs isolated from prostate cancer patients can express PSA and show molecular characteristics of prostate cancer such as *AR* copy number gain, *PTEN* loss and *TMPRSS2-ETS* gene fusion [83]. Enrichment strategies such as CellSearch are dependent on target expression, which could potentially miss CTCs that have lost the target following for example epithelial-mesenchymal transition (EMT) [84].

Microfluidic technology can enhance affinity-based capture and can capture EpCAM-independent CTCs [85, 86]. Mechanical features such as size or deformability have also been used to separate larger CTCs from other blood cells. The advantage of the latter includes that viable cells can be used for downstream analysis like CTC culture [87]. An immunofluorescence approach has also been developed showing the ability to differentiate prostate cancer CTCs based on their nuclear size and revealing that very-small-nuclear CTC (vsnCTC) correlates with visceral metastasis and could represent an aggressive variant of PC [88, 89]. Finally, Epic Sciences has clinically qualified an approach that captures all cells onto a slide and then used secondary immunofluorescence to identify and characterise CTCs. This approach may minimise cell loss but requires very high antibody specificity and sensitivity. Oncotype DX AR-V7 Nucleus Detect^®^ has been used to detect CTC expression of *AR* splice variants predicted to lack the ligand-binding domain and be constitutively active. The presence of these CTC may associate with resistance to androgen receptor signalling (ARS) inhibitors. Matched cohort studies have suggested taxanes could be preferred over ARSI when CTCs expressing *AR* splicing variants are detected, although in the absence of data from randomised clinical trials, its challenging to differentiate the effect of CTC counts and tumour volume from accurate predictions associated with the molecular marker [90, 91]. This technology can also extract other “functional” features such as CTC clusters that may have increased metastatic potential [92].

Extracellular vesicles (EV) are lipid bilayer-delimited particles containing DNA, RNA or proteins and naturally released by both normal and tumour cells. Exosomes are the most commonly studied, constituting 40–100 nm-sized particles released into blood (and other fluids) by the fusion of multivesicular bodies with the plasma membrane. They play an important role in intercellular communication, potentially contributing to tumour progression and metastases [93, 94]. Exosomes can be isolated by centrifugation, ultracentrifugation, or affinity-based capture [95, 96]. Improved extraction protocol should start to provide granularity to the clinical relevance of genetic material extracted from vesicles of various sizes [93, 97]. Several studies have already reported its potential role as a diagnostic/prognostic marker. For example, expression levels of long non-coding RNAsn (lncRNAs) isolated from exosomes showed the ability to differentiate PC from healthy donors [98]. In another study, detectable exosomal AR-V7 isolated by ultracentrifugation from plasma has been shown to associate with a shorter time to progression in CRPC [99].

miRNAs are characterised by greater stability in circulation and can be isolated directly from plasma or following EV enrichment. miRNAs are small single-stranded non-coding RNA (around 20 nucleotides) that function in post-transcriptional regulation of gene expression. Prostate cancer patients appear to have higher levels of specific miRNAs which may have distinct roles and associate with unique phenotypes [97, 100]. For example, different studies have tested the role of miR-141 and miR-375 in prostate cancer in plasma and reported association with high-risk factors of disease dissemination in localised disease and shorter time to progression in metastatic patients treated with docetaxel or abiraterone [101, 102]. Another study has shown the potential predictive role of miR-423-3P as a biomarker to identify castration resistance [103]. Clinical qualification in appropriate cohorts is now required to determine their potential role for patient management.

## Role of liquid biopsy in different clinical settings in prostate cancer

### Early detection and risk re-stratification

PSA testing is not suitable for population-based screening due to its low specificity for prostate cancer, resulting in massive over-treatment and misdiagnosis. Multiparametric magnetic resonance imaging (mpMRI) [104] has improved the current prostate cancer diagnostic pathway but distinguishing aggressive cancers that require immediate and intense treatment from cancers that do not remains challenging. A number of tissue-based molecular tests have been implemented to distinguish aggressive cancers [105, 106]. Tissue-based tests could be complemented by liquid biopsies that by identifying tumour in circulation, confirm cancer’s potential for spread.

One of the major efforts in the cancer screening space has been undertaken by GRAIL, Inc (Galleri^®^). In their Circulating Cell-free Genome Atlas (CCGA) study (designed to combine data from cfDNA sequencing and machine learning for the development of a population-based cancer screening programme) [107], they have discovered and validated a pan-cancer methylation targeted-based assay. Results were consistent between the training and validation set with a false positive rate below 1% and overall increasing sensitivity from Stage I (18%) to Stage IV (93%) for cancer detection and its tissue-of- origin (TOO). However, the sensitivity for detecting prostate cancer using the multi-cancer assay was reported to be 11.2%, which may be confounded by the inclusion of early-stage, indolent disease picked up in screening programmes [52]. It is probable that clinical assessment bespoke to prostate cancer, which is confounded both by opportunistic PSA screening and the widely variable disease course, will be required for the implementation of a prostate ctDNA screening test. As a proof-of-principle, targeted amplicon sequencing across *TP53* gene on plasma DNA samples collected from a limited number of localised prostate cancer patients before and after local treatment suggests detection of ctDNA before surgery is associated with poor clinical outcome (metastatic-free survival) [108]. Overall, a test that improves risk stratification of men at diagnosis could enable screening by correcting the error of subsequent over-treatment. This will require high sensitivity and specificity and will have to resolve the challenges of low tumour fraction, molecular heterogeneity and clonal haematopoiesis [109, 110].

ctDNA has shown promising evidence of detecting clinically relevant, minimal residual disease after primary treatment in many tumour types [11, 111, 112]. Studies in multiple cancer types have consistently shown that individuals with detectable ctDNA shortly after surgery relapsed sooner than those who were ctDNA-negative. These approaches used high-sensitivity analysis, either of recurrent mutations or patient-specific clonal mutations. Around 15% of localised prostate cancer patients eventually relapse after curative treatment [1]. However, this is often detected by a rising PSA and a ctDNA based assay would have to improve on the performance of PSA for detecting minimal residual or early recurring disease.

### Treatment response prediction and assessment in advance disease

The role of liquid biopsies is better established in advanced metastatic disease where tumour volumes and circulating tumour are higher. Moreover, the clinical need for the selection of patients for targeted treatment has followed drug development pathways with current indications focused on relapsed patients. Several companies have developed multigene targeted-capture NGS assays, intended for cfDNA analysis, covering single-nucleotide variants (SNVs), insertion or deletions (indels) and copy number alterations (CNAs) to detect a range of genomic aberrations at low allelic frequencies, primarily to provide tumour mutation profiling (and including several genes relevant for prostate cancer) [113]. More relevant to prostate cancer, is the recent FDA clearance of a liquid biopsy test for the selection of patients harbouring alterations in DNA damage repair genes, for example, the FoundationOne^®^ Liquid CDx that was developed as a companion diagnostic for the PARP inhibitors rucaparib and olaparib in metastatic castration-resistant patients (together with other cancer types). This hybridisation-based NGS panel captures 300 genes including mutations in *BRCA1* and *2*.

Olaparib was approved based on the results of the PROfound study, a randomised Phase III biomarker-driven clinical trial, in which patients progressing after abiraterone or enzalutamide received Olaparib versus the alternative ARSI [114]. Patients were stratified into two cohorts: cohort 1 (*BRCA1/2* and *ATM)* versus cohort 2 (12 others DDR genes) with positive results for radiographic PFS (rPFS) for patients included in cohort 1 (7.4 months vs. 3.6 months, HR 0.34). Rucaparib has been approved for *BRCA*-mutated patients progressing after a taxane chemotherapy and an *AR*-targeted therapy based on the results of the open-label Phase II TRITON2 study which reported and objective response rate for *BRCA1/2* aberrant of 43% [115]. Even though the benefit in *BRCA2* is well-established, given the wide range of alterations in a multitude of genes involved in homologous recombination repair, uncertainty exists for the sensitivity of specific alterations and sensitivity to PARPi, primarily as many on an individual basis are rare. Collation of outcome data, sharing across trials and continued annotation post-licensing will be important to improving prediction and patient selection.

cfDNA testing without concurrently sequencing the patient’s whole blood as control could uncommonly identify alterations in DDR genes that are presumed to be pathogenic but are clonal haematopoiesis of indeterminate potential (CHIP) variants. These false positives could lead to inappropriate treatment of patients with PARPi without the expected efficacy [116].

Different studies have also identified genomic and epigenomic features that could predict acetate abiraterone (AA) treatment response. In one study, cfDNA cytosine modification patterns from 33 prostate cancer patients treated with AA were identified and showed divergences between AA-sensitive versus AA-resistant patients and potential for predicting treatment response based on cfDNA modification variability [68]. In another study, treatment response prediction for ARSI was assessed in a pooled analysis from four CRPC cohorts, with findings of a circulating *AR* copy number of 1.92 as the cut point with the greatest association with shorter progression-free survival and overall survival on ARSI [117].

### Response assessment and outcome surrogacy

Additional to patient selection, liquid biopsies have potential for real-time tracking of treatment response and early detection of progression. For example, in studies of men with high burden relapsed metastatic prostate cancer, CTC count included in a composite panel with serum lactate dehydrogenase is a surrogate of survival and captures the effect of ARSI treatment [118]. Another study explored the CTCs dynamics in different end-points on over 6000 mCRPC patients enrolled in five Phase III clinical trials and showed its superiority in comparison to PSA responses. In fact, patients with over 1 CTC at baseline and zero at week 13 and patients with more than 5 CTCs and less than 4 at week 13, showed the longest overall survival [119]. There are also studies that have tested this question for ctDNA. For example, in a cross-over study of abiraterone vs enzalutamide in mCRPC, patients with a baseline ctDNA fraction greater than 2% were associated with worse time to PSA progression [120]. In patients responding to ARSI, samples taking while responding to therapy had a much lower ctDNA than the baseline sample [121]. Other studies are testing ctDNA and other liquid biopsy features for assessing response and preliminary data from analysis of samples after 4–8 weeks of treatment is promising for distinguishing treatment response from primary resistance [37, 39].

### Tracking of resistance

Liquid biopsies allow tracking of emerging clones on treatment. Detection of alterations may be biased by the site of metastases (for example, central nervous system metastases may not be represented) and other biological factors. Nonetheless, given most prostate cancer spread involves bone and is blood-borne, most studies have shown that tumour DNA in plasma represents clinically relevant progressing metastases. In mCRPC patients treated with PARP inhibition, ctDNA studies have identified a heterogeneous landscape of sub-clonal mutations in *BRCA2* or *PALB2* that are predicted to restore *BRCA2* function in samples collected at the progression that were not present prior to treatment [122, 123]. Both studies showed clonal divergence with several different genomic events emerging and causing resistance to PARPI. Similarly, in patients treated with abiraterone, a functionally relevant point mutation in the *AR* emerges in ~15% of progression samples [8]. Another study reported the ability to track clones over time in plasma utilising an abundance of commonly occurring deletions such as 21q22 resulting from a TMPRSS2-ERG gene fusion [7]. Tracking methylation patterns changes in ctDNA over time has also been reported to give insight about disease progression, treatment response and identify neuroendocrine markers [69, 124, 125]. Although the clinical utility of these observations has not been defined, they provide a proof-of-concept framework for interrogating molecular causes of resistance in real-time and identifying novel causes as new treatments become established.

## Future development

A fit-for-purpose liquid biopsy assay, intended for implementation in clinical practice for prostate cancer patients, should achieve three main goals: (i) analytical validation which refers to the reliability, accuracy of measure and reproducibility of the test (including the preanalytical assessment of samples and consistency across laboratories) [126], (ii) the clinical qualification which relates to a clinically meaningful biological question to be addressed in appropriately-designed clinical trials (usually first retrospectively tested in samples collected in prior studies followed by analysis in studies where the testing of the liquid biopsy is a pre-defined aim) and (iii) defining clinical utility and clinically implementation, that show use of the test improves clinical outcomes, is cost beneficial and feasible to implement [127–129].

Uncertainty remains on whether a pan-cancer or a prostate-specific test is more suitable for use in clinical practice. Firstly, blood pan-cancer tests can achieve economies of scale but may not be optimised to include prostate cancer-specific genomic alterations. Secondly, detection of gene loss and allelic imbalance at different copy number states remains challenging and requires optimised approaches. Thirdly, technological improvements can improve detection and reliability of detection of ultra-low allelic frequency variants [7, 8].

One of the main strength of blood assays is the feasibility of repeated collection which could allow earlier detection of mechanisms of resistance to inform on treatment changes or combinational therapy. Combinatorial genomic, transcriptomic and epigenomic testing on blood samples could improve the prediction of phenotype and could be integrated to maximise the potential of liquid biopsies for clinical practice.

Overall, a good prostate cancer blood assay should guide one or more clinical decision points such as diagnosis, molecular characterisation and risk stratification in an early setting, detection of early relapses, treatment cessation or intensification and identification of mechanisms of resistance to current treatments in the metastatic setting.

Addressing these challenges in the next few several years with the collaboration of clinicians, researchers and bioinformaticians, will help to understand who and when someone should be molecularly profiled, how to design better biomarker-driven clinical trials and overcome technical challenges and finally introduce the liquid biopsy in the clinical routine practice for prostate cancer patients.

## Data Availability

Data sharing is not applicable to this article, as no datasets were generated or analysed during this study.

## References

[1] CRUK. Prostate cancer mortality statistics. 2018.

[2] Cancer Genome Atlas Research N. (2015). The molecular taxonomy of primary prostate cancer. Cell.

[3] Lorente D, Omlin A, Zafeiriou Z, Nava-Rodrigues D, Perez-Lopez R, Pezaro C (2016). Castration-resistant prostate cancer tissue acquisition from bone metastases for molecular analyses. Clin Genitourin Cancer.

[4] Lo YM, Corbetta N, Chamberlain PF, Rai V, Sargent IL, Redman CW (1997). Presence of fetal DNA in maternal plasma and serum. Lancet..

[5] Yu SC, Chan KC, Zheng YW, Jiang P, Liao GJ, Sun H (2014). Size-based molecular diagnostics using plasma DNA for noninvasive prenatal testing. Proc Natl Acad Sci USA.

[6] Diehl F, Li M, Dressman D, He Y, Shen D, Szabo S (2005). Detection and quantification of mutations in the plasma of patients with colorectal tumors. Proc Natl Acad Sci USA.

[7] Carreira S, Romanel A, Goodall J, Grist E, Ferraldeschi R, Miranda S (2014). Tumor clone dynamics in lethal prostate cancer. Sci Transl Med.

[8] Romanel A, Gasi Tandefelt D, Conteduca V, Jayaram A, Casiraghi N, Wetterskog D (2015). Plasma AR and abiraterone-resistant prostate cancer. Sci Transl Med.

[9] Haber DA, Velculescu VE (2014). Blood-based analyses of cancer: circulating tumor cells and circulating tumor DNA. Cancer Discov.

[10] Forshew T, Murtaza M, Parkinson C, Gale D, Tsui DW, Kaper F (2012). Noninvasive identification and monitoring of cancer mutations by targeted deep sequencing of plasma DNA. Sci Transl Med.

[11] Abbosh C, Birkbak NJ, Wilson GA, Jamal-Hanjani M, Constantin T, Salari R (2017). Phylogenetic ctDNA analysis depicts early-stage lung cancer evolution. Nature..

[12] Cimmino I, Bravaccini S, Cerchione C (2021). Urinary biomarkers in tumors: an overview. Methods Mol Biol.

[13] Casadio V, Salvi S, Martignano F, Gunelli R, Ravaioli S, Calistri D, et al. Integrity analysis in urine samples. J Vis Exp. 2017;119:e55049. 10.3791/55049 PMC5408646 28117781

[14] Rigau M, Olivan M, Garcia M, Sequeiros T, Montes M, Colas E (2013). The present and future of prostate cancer urine biomarkers. Int J Mol Sci.

[15] de Kok JB, Hendriks JC, van Solinge WW, Willems HL, Mensink EJ, Swinkels DW (1998). Use of real-time quantitative PCR to compare DNA isolation methods. Clin Chem.

[16] Chiu RW, Poon LL, Lau TK, Leung TN, Wong EM, Lo YM (2001). Effects of blood-processing protocols on fetal and total DNA quantification in maternal plasma. Clin Chem.

[17] Jung M, Klotzek S, Lewandowski M, Fleischhacker M, Jung K (2003). Changes in concentration of DNA in serum and plasma during storage of blood samples. Clin Chem.

[18] Wong D, Moturi S, Angkachatchai V, Mueller R, DeSantis G, van den Boom D (2013). Optimizing blood collection, transport and storage conditions for cell free DNA increases access to prenatal testing. Clin Biochem.

[19] Beranek M, Sirak I, Vosmik M, Petera J, Drastikova M, Palicka V (2016). Carrier molecules and extraction of circulating tumor DNA for next generation sequencing in colorectal cancer. Acta Med.

[20] Qin J, Alt JR, Hunsley BA, Williams TL, Fernando MR (2014). Stabilization of circulating tumor cells in blood using a collection device with a preservative reagent. Cancer Cell Int.

[21] Stefansson S, Adams DL, Ershler WB, Le H, Ho DH (2016). A cell transportation solution that preserves live circulating tumor cells in patient blood samples. BMC Cancer.

[22] Zong C, Lu S, Chapman AR, Xie XS (2012). Genome-wide detection of single-nucleotide and copy-number variations of a single human cell. Science.

[23] Treff NR, Su J, Tao X, Northrop LE, Scott RT (2011). Single-cell whole-genome amplification technique impacts the accuracy of SNP microarray-based genotyping and copy number analyses. Mol Hum Reprod.

[24] Baek R, Sondergaard EK, Varming K, Jorgensen MM (2016). The impact of various preanalytical treatments on the phenotype of small extracellular vesicles in blood analyzed by protein microarray. J Immunol Methods.

[25] Mullier F, Bailly N, Chatelain C, Chatelain B, Dogne JM (2013). Pre-analytical issues in the measurement of circulating microparticles: current recommendations and pending questions. J Thromb Haemost.

[26] Torrano V, Royo F, Peinado H, Loizaga-Iriarte A, Unda M, Falcon-Perez JM (2016). Vesicle-MaNiA: extracellular vesicles in liquid biopsy and cancer. Curr Opin Pharm.

[27] Thery C, Amigorena S, Raposo G, Clayton A. Isolation and characterization of exosomes from cell culture supernatants and biological fluids. Curr Protoc Cell Biol. 2006;Chapter 3:Unit 3 22. 10.1002/0471143030.cb0322s30 18228490

[28] Lotvall J, Hill AF, Hochberg F, Buzas EI, Di Vizio D, Gardiner C (2014). Minimal experimental requirements for definition of extracellular vesicles and their functions: a position statement from the International Society for Extracellular Vesicles. J Extracell Vesicles.

[29] Wyatt AW, Annala M, Aggarwal R, Beja K, Feng F, Youngren J, et al. Concordance of circulating tumor DNA and matched metastatic tissue biopsy in prostate cancer. J Natl Cancer Inst. 2017;109. 10.1093/jnci/djx118 PMC6440274 29206995

[30] Vandekerkhove G, Struss WJ, Annala M, Kallio HML, Khalaf D, Warner EW (2019). Circulating tumor DNA abundance and potential utility in de novo metastatic prostate cancer. Eur Urol.

[31] Odegaard JI, Vincent JJ, Mortimer S, Vowles JV, Ulrich BC, Banks KC (2018). Validation of a plasma-based comprehensive cancer genotyping assay utilizing orthogonal tissue- and plasma-based methodologies. Clin Cancer Res.

[32] Pritchard CC, Salipante SJ, Koehler K, Smith C, Scroggins S, Wood B (2014). Validation and implementation of targeted capture and sequencing for the detection of actionable mutation, copy number variation, and gene rearrangement in clinical cancer specimens. J Mol Diagn.

[33] Woodhouse R, Li M, Hughes J, Delfosse D, Skoletsky J, Ma P (2020). Clinical and analytical validation of FoundationOne Liquid CDx, a novel 324-Gene cfDNA-based comprehensive genomic profiling assay for cancers of solid tumor origin. PLoS ONE.

[34] Kingston B, Cutts RJ, Bye H, Beaney M, Walsh-Crestani G, Hrebien S (2021). Genomic profile of advanced breast cancer in circulating tumour DNA. Nat Commun.

[35] Russo M, Siravegna G, Blaszkowsky LS, Corti G, Crisafulli G, Ahronian LG (2016). Tumor heterogeneity and lesion-specific response to targeted therapy in colorectal cancer. Cancer Discov..

[36] Jamal-Hanjani M, Wilson GA, McGranahan N, Birkbak NJ, Watkins TBK, Veeriah S (2017). Tracking the evolution of non-small-cell lung cancer. N. Engl J Med.

[37] Jayaram A, Wingate A, Wetterskog D, Wheeler G, Sternberg CN, Jones R (2021). Plasma tumor gene conversions after one cycle abiraterone acetate for metastatic castration-resistant prostate cancer: a biomarker analysis of a multicenter international trial. Ann Oncol.

[38] Heitzer E, Ulz P, Belic J, Gutschi S, Quehenberger F, Fischereder K (2013). Tumor-associated copy number changes in the circulation of patients with prostate cancer identified through whole-genome sequencing. Genome Med.

[39] Conteduca V, Wetterskog D, Scarpi E, Romanel A, Gurioli G, Jayaram A (2020). Plasma tumour DNA as an early indicator of treatment response in metastatic castration-resistant prostate cancer. Br J Cancer.

[40] Robinson D, Van Allen EM, Wu YM, Schultz N, Lonigro RJ, Mosquera JM (2015). Integrative clinical genomics of advanced prostate. Cancer Cell..

[41] Prandi D, Baca SC, Romanel A, Barbieri CE, Mosquera JM, Fontugne J (2014). Unraveling the clonal hierarchy of somatic genomic aberrations. Genome Biol.

[42] Conteduca V, Wetterskog D, Sharabiani MTA, Grande E, Fernandez-Perez MP, Jayaram A (2017). Androgen receptor gene status in plasma DNA associates with worse outcome on enzalutamide or abiraterone for castration-resistant prostate cancer: a multi-institution correlative biomarker study. Ann Oncol.

[43] Annala M, Vandekerkhove G, Khalaf D, Taavitsainen S, Beja K, Warner EW (2018). Circulating tumor DNA genomics correlate with resistance to abiraterone and enzalutamide in prostate. Cancer Cancer Discov..

[44] Pritchard CC, Mateo J, Walsh MF, De Sarkar N, Abida W, Beltran H (2016). Inherited DNA-repair gene mutations in men with metastatic prostate cancer. N. Engl J Med.

[45] Hussain M, Mateo J, Fizazi K, Saad F, Shore N, Sandhu S (2020). Survival with olaparib in metastatic castration-resistant prostate cancer. N. Engl J Med.

[46] Abida W, Cheng ML, Armenia J, Middha S, Autio KA, Vargas HA (2019). Analysis of the prevalence of microsatellite instability in prostate cancer and response to immune checkpoint blockade. JAMA Oncol.

[47] Aggarwal RR, Quigley DA, Huang J, Zhang L, Beer TM, Rettig MB (2019). Whole-genome and transcriptional analysis of treatment-emergent small-cell neuroendocrine prostate cancer demonstrates intraclass heterogeneity. Mol Cancer Res.

[48] Egger G, Liang G, Aparicio A, Jones PA (2004). Epigenetics in human disease and prospects for epigenetic therapy. Nature..

[49] Lister R, Pelizzola M, Dowen RH, Hawkins RD, Hon G, Tonti-Filippini J (2009). Human DNA methylomes at base resolution show widespread epigenomic differences. Nature..

[50] Luo H, Zhao Q, Wei W, Zheng L, Yi S, Li G, et al. Circulating tumor DNA methylation profiles enable early diagnosis, prognosis prediction, and screening for colorectal cancer. Sci Transl Med. 2020;12:eaax7533. 10.1126/scitranslmed.aax7533 31894106

[51] Xu RH, Wei W, Krawczyk M, Wang W, Luo H, Flagg K (2017). Circulating tumour DNA methylation markers for diagnosis and prognosis of hepatocellular carcinoma. Nat Mater.

[52] Klein EA, Richards D, Cohn A, Tummala M, Lapham R, Cosgrove D (2021). Clinical validation of a targeted methylation-based multi-cancer early detection test using an independent validation set. Ann Oncol.

[53] Chakravarthy A, Furness A, Joshi K, Ghorani E, Ford K, Ward MJ (2018). Pan-cancer deconvolution of tumour composition using DNA methylation. Nat Commun.

[54] Moss J, Magenheim J, Neiman D, Zemmour H, Loyfer N, Korach A (2018). Comprehensive human cell-type methylation atlas reveals origins of circulating cell-free DNA in health and disease. Nat Commun.

[55] Teschendorff AE, Relton CL (2018). Statistical and integrative system-level analysis of DNA methylation data. Nat Rev Genet.

[56] Friedlander TW, Roy R, Tomlins SA, Ngo VT, Kobayashi Y, Azameera A (2012). Common structural and epigenetic changes in the genome of castration-resistant prostate cancer. Cancer Res.

[57] Roupret M, Hupertan V, Catto JW, Yates DR, Rehman I, Proctor LM (2008). Promoter hypermethylation in circulating blood cells identifies prostate cancer progression. Int J Cancer.

[58] Paziewska A, Dabrowska M, Goryca K, Antoniewicz A, Dobruch J, Mikula M (2014). DNA methylation status is more reliable than gene expression at detecting cancer in prostate biopsy. Br J Cancer.

[59] Kang S, Li Q, Chen Q, Zhou Y, Park S, Lee G (2017). CancerLocator: non-invasive cancer diagnosis and tissue-of-origin prediction using methylation profiles of cell-free DNA. Genome Biol.

[60] Li W, Li Q, Kang S, Same M, Zhou Y, Sun C (2018). CancerDetector: ultrasensitive and non-invasive cancer detection at the resolution of individual reads using cell-free DNA methylation sequencing data. Nucleic Acids Res.

[61] Wang J, Yang L, Diao Y, Liu J, Li J, Li R (2021). Circulating tumour DNA methylation in hepatocellular carcinoma diagnosis using digital droplet PCR. J Int Med Res.

[62] Massie CE, Mills IG, Lynch AG (2017). The importance of DNA methylation in prostate cancer development. J Steroid Biochem Mol Biol.

[63] Henrique R, Jeronimo C (2004). Molecular detection of prostate cancer: a role for GSTP1 hypermethylation. Eur Urol.

[64] Mian OY, Khattab MH, Hedayati M, Coulter J, Abubaker-Sharif B, Schwaninger JM (2016). GSTP1 Loss results in accumulation of oxidative DNA base damage and promotes prostate cancer cell survival following exposure to protracted oxidative stress. Prostate..

[65] Maldonado L, Brait M, Loyo M, Sullenberger L, Wang K, Peskoe SB (2014). GSTP1 promoter methylation is associated with recurrence in early stage prostate cancer. J Urol.

[66] Mahon KL, Qu W, Devaney J, Paul C, Castillo L, Wykes RJ (2014). Methylated glutathione S-transferase 1 (mGSTP1) is a potential plasma free DNA epigenetic marker of prognosis and response to chemotherapy in castrate-resistant prostate cancer. Br J Cancer.

[67] Hendriks RJ, Dijkstra S, Smit FP, Vandersmissen J, Van de Voorde H, Mulders PFA (2018). Epigenetic markers in circulating cell-free DNA as prognostic markers for survival of castration-resistant prostate cancer patients. Prostate..

[68] Gordevicius J, Krisciunas A, Groot DE, Yip SM, Susic M, Kwan A (2018). Cell-free DNA modification dynamics in abiraterone acetate-treated prostate cancer patients. Clin Cancer Res.

[69] Silva R, Moran B, Baird AM, O’Rourke CJ, Finn SP, McDermott R (2021). Longitudinal analysis of individual cfDNA methylome patterns in metastatic prostate cancer. Clin Epigenetics.

[70] Wu A, Cremaschi P, Wetterskog D, Conteduca V, Franceschini GM, Kleftogiannis D (2020). Genome-wide plasma DNA methylation features of metastatic prostate cancer. J Clin Investig..

[71] Li W, Zhang X, Lu X, You L, Song Y, Luo Z (2017). 5-Hydroxymethylcytosine signatures in circulating cell-free DNA as diagnostic biomarkers for human cancers. Cell Res.

[72] Beltran H, Romanel A, Conteduca V, Casiraghi N, Sigouros M, Franceschini GM (2020). Circulating tumor DNA profile recognizes transformation to castration-resistant neuroendocrine prostate cancer. J Clin Investig..

[73] Snyder MW, Kircher M, Hill AJ, Daza RM, Shendure J, Cell-free DNA (2016). Comprises an in vivo nucleosome footprint that informs its tissues-of-origin. Cell.

[74] Sun K, Jiang P, Cheng SH, Cheng THT, Wong J, Wong VWS (2019). Orientation-aware plasma cell-free DNA fragmentation analysis in open chromatin regions informs tissue of origin. Genome Res.

[75] Ulz P, Perakis S, Zhou Q, Moser T, Belic J, Lazzeri I (2019). Inference of transcription factor binding from cell-free DNA enables tumor subtype prediction and early detection. Nat Commun.

[76] Jiang P, Chan CW, Chan KC, Cheng SH, Wong J, Wong VW (2015). Lengthening and shortening of plasma DNA in hepatocellular carcinoma patients. Proc Natl Acad Sci USA.

[77] Yamamoto Y, Uemura M, Fujita M, Maejima K, Koh Y, Matsushita M (2019). Clinical significance of the mutational landscape and fragmentation of circulating tumor DNA in renal cell carcinoma. Cancer Sci.

[78] Underhill HR, Kitzman JO, Hellwig S, Welker NC, Daza R, Baker DN (2016). Fragment length of circulating tumor DNA. PLoS Genet.

[79] Mouliere F, Chandrananda D, Piskorz AM, Moore EK, Morris J, Ahlborn LB, et al. Enhanced detection of circulating tumor DNA by fragment size analysis. Sci Transl Med. 2018;10:eaat4921. 10.1126/scitranslmed.aat4921 PMC6483061 30404863

[80] Pantel K, Alix-Panabieres C (2016). Functional studies on viable circulating tumor cells. Clin Chem.

[81] Kanwar N, Hu P, Bedard P, Clemons M, McCready D, Done SJ (2015). Identification of genomic signatures in circulating tumor cells from breast cancer. Int J Cancer.

[82] de Bono JS, Scher HI, Montgomery RB, Parker C, Miller MC, Tissing H (2008). Circulating tumor cells predict survival benefit from treatment in metastatic castration-resistant prostate cancer. Clin Cancer Res.

[83] Attard G, Swennenhuis JF, Olmos D, Reid AH, Vickers E, A’Hern R (2009). Characterization of ERG, AR and PTEN gene status in circulating tumor cells from patients with castration-resistant prostate cancer. Cancer Res.

[84] Kalluri R, Weinberg RA (2009). The basics of epithelial-mesenchymal transition. J Clin Investig.

[85] Ozkumur E, Shah AM, Ciciliano JC, Emmink BL, Miyamoto DT, Brachtel E (2013). Inertial focusing for tumor antigen-dependent and -independent sorting of rare circulating tumor cells. Sci Transl Med.

[86] Gleghorn JP, Pratt ED, Denning D, Liu H, Bander NH, Tagawa ST (2010). Capture of circulating tumor cells from whole blood of prostate cancer patients using geometrically enhanced differential immunocapture (GEDI) and a prostate-specific antibody. Lab Chip.

[87] Xu L, Mao X, Imrali A, Syed F, Mutsvangwa K, Berney D (2015). Optimization and evaluation of a novel size based circulating tumor cell isolation system. PLoS ONE.

[88] Chen JF, Ho H, Lichterman J, Lu YT, Zhang Y, Garcia MA (2015). Subclassification of prostate cancer circulating tumor cells by nuclear size reveals very small nuclear circulating tumor cells in patients with visceral metastases. Cancer..

[89] Beltran H, Tomlins S, Aparicio A, Arora V, Rickman D, Ayala G (2014). Aggressive variants of castration-resistant prostate cancer. Clin Cancer Res.

[90] Antonarakis ES, Lu C, Wang H, Luber B, Nakazawa M, Roeser JC (2014). AR-V7 and resistance to enzalutamide and abiraterone in prostate cancer. N. Engl J Med.

[91] Scher HI, Graf RP, Schreiber NA, Jayaram A, Winquist E, McLaughlin B (2018). Assessment of the validity of nuclear-localized androgen receptor splice variant 7 in circulating tumor cells as a predictive biomarker for castration-resistant prostate cancer. JAMA Oncol.

[92] Beltran H, Jendrisak A, Landers M, Mosquera JM, Kossai M, Louw J (2016). The initial detection and partial characterization of circulating tumor cells in neuroendocrine prostate cancer. Clin Cancer Res.

[93] Liu CM, Hsieh CL, Shen CN, Lin CC, Shigemura K, Sung SY (2016). Exosomes from the tumor microenvironment as reciprocal regulators that enhance prostate cancer progression. Int J Urol.

[94] Li FX, Liu JJ, Xu F, Lin X, Zhong JY, Wu F (2019). Role of tumor-derived exosomes in bone metastasis. Oncol Lett.

[95] Lobb RJ, Becker M, Wen SW, Wong CS, Wiegmans AP, Leimgruber A (2015). Optimized exosome isolation protocol for cell culture supernatant and human plasma. J Extracell Vesicles.

[96] Zeringer E, Barta T, Li M, Vlassov AV (2015). Strategies for isolation of exosomes. Cold Spring Harb Protoc.

[97] Pan J, Ding M, Xu K, Yang C, Mao LJ (2017). Exosomes in diagnosis and therapy of prostate cancer. Oncotarget..

[98] Wang YH, Ji J, Wang BC, Chen H, Yang ZH, Wang K (2018). Tumor-derived exosomal long noncoding RNAs as promising diagnostic biomarkers for prostate cancer. Cell Physiol Biochem.

[99] Joncas FH, Lucien F, Rouleau M, Morin F, Leong HS, Pouliot F (2019). Plasma extracellular vesicles as phenotypic biomarkers in prostate cancer patients. Prostate..

[100] Porzycki P, Ciszkowicz E, Semik M, Tyrka M (2018). Combination of three miRNA (miR-141, miR-21, and miR-375) as potential diagnostic tool for prostate cancer recognition. Int Urol Nephrol.

[101] Brase JC, Johannes M, Schlomm T, Falth M, Haese A, Steuber T (2011). Circulating miRNAs are correlated with tumor progression in prostate cancer. Int J Cancer.

[102] Zedan AH, Osther PJS, Assenholt J, Madsen JS, Hansen TF (2020). Circulating miR-141 and miR-375 are associated with treatment outcome in metastatic castration resistant prostate cancer. Sci Rep..

[103] Guo T, Wang Y, Jia J, Mao X, Stankiewicz E, Scandura G (2020). The identification of plasma exosomal miR-423-3p as a potential predictive biomarker for prostate cancer castration-resistance development by plasma exosomal miRNA sequencing. Front Cell Dev Biol.

[104] Ahmed HU, El-Shater Bosaily A, Brown LC, Gabe R, Kaplan R, Parmar MK (2017). Diagnostic accuracy of multi-parametric MRI and TRUS biopsy in prostate cancer (PROMIS): a paired validating confirmatory study. Lancet..

[105] Spratt DE, Yousefi K, Deheshi S, Ross AE, Den RB, Schaeffer EM (2017). Individual patient-level meta-analysis of the performance of the decipher genomic classifier in high-risk men after prostatectomy to predict development of metastatic disease. J Clin Oncol.

[106] Klein EA, Cooperberg MR, Magi-Galluzzi C, Simko JP, Falzarano SM, Maddala T (2014). A 17-gene assay to predict prostate cancer aggressiveness in the context of Gleason grade heterogeneity, tumor multifocality, and biopsy undersampling. Eur Urol.

[107] Liu MC, Oxnard GR, Klein EA, Swanton C, Seiden MV, Consortium C (2020). Sensitive and specific multi-cancer detection and localization using methylation signatures in cell-free DNA. Ann Oncol.

[108] Lau E, McCoy P, Reeves F, Chow K, Clarkson M, Kwan EM (2020). Detection of ctDNA in plasma of patients with clinically localised prostate cancer is associated with rapid disease progression. Genome Med.

[109] Hennigan ST, Trostel SY, Terrigino NT, Voznesensky OS, Schaefer RJ, Whitlock NC, et al. Low abundance of circulating tumor DNA in localized prostate cancer. JCO Precis Oncol. 2019;3:1–13. 10.1200/PO.19.00176 PMC6746181 31528835

[110] Mayrhofer M, De Laere B, Whitington T, Van Oyen P, Ghysel C, Ampe J (2018). Cell-free DNA profiling of metastatic prostate cancer reveals microsatellite instability, structural rearrangements and clonal hematopoiesis. Genome Med.

[111] Reinert T, Henriksen TV, Christensen E, Sharma S, Salari R, Sethi H (2019). Analysis of plasma cell-free DNA by ultradeep sequencing in patients with stages I to III colorectal cancer. JAMA Oncol.

[112] Coombes RC, Page K, Salari R, Hastings RK, Armstrong A, Ahmed S (2019). Personalized detection of circulating tumor DNA antedates breast cancer metastatic recurrence. Clin Cancer Res.

[113] Scheerens H, Malong A, Bassett K, Boyd Z, Gupta V, Harris J (2017). Current status of companion and complementary diagnostics: strategic considerations for development and launch. Clin Transl Sci.

[114] de Bono J, Mateo J, Fizazi K, Saad F, Shore N, Sandhu S (2020). Olaparib for metastatic castration-resistant prostate cancer. N. Engl J Med.

[115] Abida W, Patnaik A, Campbell D, Shapiro J, Bryce AH, McDermott R (2020). Rucaparib in men with metastatic castration-resistant prostate cancer harboring a BRCA1 or BRCA2 gene alteration. J Clin Oncol.

[116] Jensen K, Konnick EQ, Schweizer MT, Sokolova AO, Grivas P, Cheng HH (2021). Association of clonal hematopoiesis in DNA repair genes with prostate cancer plasma cell-free DNA testing interference. JAMA Oncol.

[117] Jayaram A, Wingate A, Wetterskog D, Conteduca V, Khalaf D, Sharabiani MTA, et al. Plasma androgen receptor copy number status at emergence of metastatic castration-resistant prostate cancer: a pooled multicohort analysis. JCO Precis Oncol. 2019;3:1–13. 10.1200/PO.19.00123 PMC7446348 32923850

[118] Scher HI, Heller G, Molina A, Attard G, Danila DC, Jia X (2015). Circulating tumor cell biomarker panel as an individual-level surrogate for survival in metastatic castration-resistant prostate cancer. J Clin Oncol.

[119] Heller G, McCormack R, Kheoh T, Molina A, Smith MR, Dreicer R (2018). Circulating tumor cell number as a response measure of prolonged survival for metastatic castration-resistant prostate cancer: a comparison with prostate-specific antigen across five randomized phase III clinical trials. J Clin Oncol.

[120] Chi KN, Annala M, Sunderland K, Khalaf D, Finch D, Oja CD (2017). A randomized phase II cross-over study of abiraterone + prednisone (ABI) vs enzalutamide (ENZ) for patients (pts) with metastatic, castration-resistant prostate cancer (mCRPC). J Clin Oncol.

[121] Wyatt AW, Azad AA, Volik SV, Annala M, Beja K, McConeghy B (2016). Genomic alterations in cell-free DNA and enzalutamide resistance in castration-resistant prostate cancer. JAMA Oncol.

[122] Quigley D, Alumkal JJ, Wyatt AW, Kothari V, Foye A, Lloyd P (2017). Analysis of circulating cell-free DNA identifies multiclonal heterogeneity of BRCA2 reversion mutations associated with resistance to PARP inhibitors. Cancer Discov.

[123] Goodall J, Mateo J, Yuan W, Mossop H, Porta N, Miranda S (2017). Circulating cell-free DNA to guide prostate cancer treatment with PARP inhibition. Cancer Discov.

[124] Peter MR, Bilenky M, Isserlin R, Bader GD, Shen SY, De Carvalho DD (2020). Dynamics of the cell-free DNA methylome of metastatic prostate cancer during androgen-targeting treatment. Epigenomics..

[125] Mahon KL, Qu W, Lin HM, Spielman C, Cain D, Jacobs C (2019). Serum free methylated glutathione S-transferase 1 DNA levels, survival, and response to docetaxel in metastatic, castration-resistant prostate cancer: post hoc analyses of data from a phase 3 trial. Eur Urol.

[126] Deveson IW, Gong B, Lai K, LoCoco JS, Richmond TA, Schageman J (2021). Evaluating the analytical validity of circulating tumor DNA sequencing assays for precision oncology. Nat Biotechnol.

[127] Teutsch SM, Bradley LA, Palomaki GE, Haddow JE, Piper M, Calonge N (2009). The evaluation of genomic applications in practice and prevention (EGAPP) initiative: methods of the EGAPP working group. Genet Med.

[128] Scher HI, Morris MJ, Larson S, Heller G (2013). Validation and clinical utility of prostate cancer biomarkers. Nat Rev Clin Oncol.

[129] Parkinson DR, McCormack RT, Keating SM, Gutman SI, Hamilton SR, Mansfield EA (2014). Evidence of clinical utility: an unmet need in molecular diagnostics for patients with cancer. Clin Cancer Res.

